# Modeling of suitable geographic areas for *Striacosta albicosta* in corn and dry bean crops under climate change scenarios

**DOI:** 10.1002/ps.70270

**Published:** 2025-09-29

**Authors:** Poliana S Pereira, Julie A Peterson, Rodrigo S Ramos, Katharine A Swoboda Bhattarai, Marcelo C Picanço, Renato A Sarmento

**Affiliations:** ^1^ Postgraduate Program in Plant Production Federal University of Tocantins Gurupi Brazil; ^2^ Department of Entomology, University of Nebraska‐Lincoln West Central Research, Extension & Education Center North Platte Nebraska USA; ^3^ Department of Entomology Federal University of Viçosa Viçosa Brazil

**Keywords:** Western bean cutworm (WBC), *Zea mays*, *Phaseolus vulgaris*, MaxEnt, climate modeling

## Abstract

**BACKGROUND:**

*Striacosta albicosta* (Lepidoptera: Noctuidae) is an important pest that causes damage to corn and dry beans. This pest originally occurred only in parts of the western United States, but initiated a concerning range expansion in 1999 and is now present in eastern North America, particularly in the Great Lakes region. Consequently, evaluating the geographical distribution of this insect is very important for its management, as it helps to identify current occurrences and predict future spread into different regions of the world. We investigated areas suitable for the establishment of the pest *S. albicosta* and its hosts, under current and future climate scenarios.

**RESULTS:**

The variables that contributed most to the model were the mean annual temperature range, mean annual temperature, and precipitation of the driest month. Although the pest is currently restricted to North America, the study indicates that regions in Europe, Asia, Oceania, South America, and Africa also present suitable conditions for its occurrence. Under current conditions, 11.41% of the area was classified as suitable, whereas 8.81% was identified as highly suitable for *S. albicosta*.

**CONCLUSION:**

This study is the first to identify regions with suitable climatic conditions for the introduction and establishment of this pest under current and future climate scenarios. These results can guide government agencies in implementing preventive measures, such as inspections and quarantines, to prevent the spread of this pest to new areas. © 2025 The Author(s). *Pest Management Science* published by John Wiley & Sons Ltd on behalf of Society of Chemical Industry.

## Graphical abstract

In recent years, the occurrence of the pest *Striacosta albicosta* has expanded from western to eastern North America. This study identified areas with suitable climatic conditions for the establishment of this pest. Our findings may guide government agencies to prevent the spread of this pest to new areas.

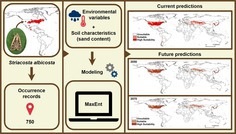

## 1 INTRODUCTION

Pests can spread to different regions of the planet through natural or anthropic dispersion, provided that suitable hosts and favorable climatic conditions are available. Climate, therefore, significantly affects the distribution and abundance of arthropod species. Consequently, predicted climate change is expected to significantly impact insect populations, both in natural ecosystems and agroecosystems.^1^, ^2^

Understanding the geographical distribution of insect pests is of great importance for the development of policies, strategies and actions to prevent their introduction or propagation into new areas.^3^, ^4^

Species Distribution Models (SDMs), such as MaxEnt, are commonly used to predict pest distribution.^2^ SDM is a tool that uses environmental variables—such as temperature, precipitation, soil conditions, and humidity—of the locations where the species already occurs to estimate areas that present favorable conditions for the species' survival and development.^5^ MaxEnt utilizes known geographical distributions to infer the climate response relationships of a species. It then projects the likelihood of introduction of the species into other regions of the world, under current climate conditions and future climatic scenarios.^1^, ^2^, ^6^

The western bean cutworm, *Striacosta albicosta* (Smith; Lepidoptera: Noctuidae), is a harmful pest in corn (*Zea mays* L.) and dry bean (*Phaseolus vulgaris* L.) crops. The larvae feed on the reproductive parts of plants, resulting in significant losses in both quantity and quality of the yield. Yield losses of ≤40% in corn^7^, ^8^ and ≤10% in dry beans have been reported.^8^ Furthermore, damage caused by larval feeding also can lead to secondary fungal infections in corn ears or expose pods to pathogens and other insects in dry beans.^9^

Historically, *S. albicosta* was limited to specific regions of the western Great Plains in the United States, including western Nebraska and Kansas, Idaho and eastern Colorado. However, in the early 2000s, populations of this species began to expand eastward into the U.S. Corn Belt, Texas, New York, Canada and Mexico.^10^, ^11^, ^12^ Hypothesized causes for this sudden range expansion have included a combination of ecological factors (such as climate, resource availability and the absence of natural enemies) and agronomic factors [such as an increase in cultivated areas, anthropogenic dispersal (transport of contaminated material), and management practices].^13^ The expanding geographical range of this insect, and its documented resistance to the Cry1F Bt protein, has become a significant concern for many producers.^14^ Consequently, research and extension projects are being performed to develop management programs in Nebraska and other regions.^7^, ^15^, ^16^

Despite the importance of this pest and its recent range expansion, no study has yet mapped the regions most suitable for its occurrence. This work represents the first effort on modeling the potential distribution of *S. albicosta* and its hosts (*Z. mays* and *P. vulgaris*) under current and future climatic scenarios using MaxEnt.

## 2 MATERIALS AND METHODS

### 2.1 Species occurrence

Occurrence records of *S. albicosta* were obtained from publicly accessible data from the Global Biodiversity Information Facility,^17^ Great Lakes and Maritimes Pest Monitoring Network,^18^ Butterflies and Moths of North America,^19^ peer‐reviewed publications,^11^, ^12^, ^14^, ^15^, ^20^, ^21^, ^22^, ^23^, ^24^, ^25^, ^26^, ^27^, ^28^ university extension reports,^29^, ^30^, ^31^, ^32^, ^33^, ^34^, ^35^, ^36^, ^37^ state and federal agricultural agency reports,^38^, ^39^, ^40^ and through personal communication with researchers who conduct pest‐trapping (K. Hamby, T. Hunt, D. Owens, R. Wright, and S. Zukoff communication with J. Peterson). Geographical locations for the occurrence of *Z. mays* and *P. vulgaris* (the primary host plants of *S. albicosta*) were collected from the Global Biodiversity Information Facility^17^ and the USDA.^41^

We confirmed that all species occurrence points were obtained from open‐field observations, for a total of 750 records for *S. albicosta*, 796 for *Z. mays* and 365 for *P. vulgaris*. The occurrence points for *S. albicosta* were exclusively within North America, whereas the two crop plant species occurred globally. All species occurrence points were filtered through spthin, an R software package (v3.5.0), to ensure that each cell can have only one occurrence within the 5‐km resolution level used by the environmental data in the models.^1^, ^42^, ^43^ After filtering, the points of occurrence for *S. albicosta* were reduced to 482, *Z. mays* to 650 and *P. vulgaris* to 362.

### 2.2 Environmental data

We initially considered 20 common environmental parameters, such as temperature, precipitation, seasonality, and soil characteristics (‘bio1’ through ‘bio20’ listed in Table 1). Nineteen of these parameters were obtained from the WorldClim dataset (v2; http://www.worldclim.org/bioclim)^1^, ^44^; and one parameter (sand content mass fraction at a 5 cm depth) was obtained from the International Soil Reference and Information Center (ISRIC; https://www.isric.org/geonetwork).^45^ This variable was included because it is important for the development of the pest's life cycle, as the prepupal and pupal stages occur in the soil.^12^, ^15^ A spatial resolution of 2.5 min (±5 km) was chosen as this is considered a quality resolution to support climate variables on a global scale.^44^

**Table 1:** Cross‐correlation (Pearson correlation coefficient, *r*) among environmental variables. (Model: *Striacosta albicosta, Zea mays* and *Phaseolus vulgaris*)

	**bio1**	bio2	bio3	bio4	bio5	bio6	bio7	bio8	bio9	bio10	bio11	bio12	bio13	bio14	bio15	bio16	bio17	bio18	bio19
**bio2**	0.463																		
bio3	0.817	0.308																	
bio4	−0.808	−0.119	−0.884																
bio5	0.886	0.673	0.567	−0.455															
bio6	0.964	0.280	0.876	−0.924	0.742														
**bio7**	−0.695	0.118	−0.826	0.968	−0.289	−0.857													
bio8	0.803	0.483	0.593	−0.456	0.843	0.688	−0.333												
bio9	0.940	0.379	0.792	−0.840	0.781	0.948	−0.752	0.597											
bio10	0.932	0.580	0.623	−0.541	0.987	0.812	−0.400	0.862	0.831										
bio11	0.978	0.358	0.878	−0.912	0.776	0.996	−0.823	0.716	0.950	0.838									
**bio12**	0.397	−0.242	0.581	−0.566	0.138	0.505	−0.615	0.261	0.385	0.218	0.473								
bio13	0.456	−0.118	0.576	−0.567	0.233	0.531	−0.578	0.357	0.412	0.303	0.515	0.897							
**bio14**	0.111	−0.329	0.279	−0.279	−0.077	0.216	−0.367	−0.0004	0.144	−0.013	0.173	0.725	0.415						
**bio15**	0.329	0.466	0.243	−0.156	0.386	0.232	−0.035	0.404	0.218	0.369	0.282	−0.151	0.165	−0.507					
bio16	0.452	−0.133	0.582	−0.572	0.222	0.531	−0.587	0.346	0.411	0.293	0.513	0.923	0.993	0.451	0.123				
bio17	0.133	−0.332	0.308	−0.307	−0.065	0.242	−0.394	−0.014	0.166	−0.002	0.198	0.757	0.449	0.994	−0.505	0.485			
bio18	0.247	−0.195	0.377	−0.358	0.049	0.308	−0.404	0.265	0.169	0.122	0.291	0.804	0.744	0.575	−0.081	0.763	0.597		
bio19	0.259	−0.240	0.443	−0.418	0.061	0.369	−0.479	0.073	0.313	0.119	0.329	0.760	0.599	0.667	−0.263	0.627	0.693	0.383	
**bio20**	0.357	0.320	0.265	0.299	0.328	0.327	−0.233	0.252	0.352	0.332	0.352	−0.186	−0.151	−0.189	0.243	0.154	−0.194	−0.230	−0.126

*Note*: Bold font indicates variables in the final model. bio1 = annual mean temperature; bio2 = mean diurnal range (mean of monthly: maximum temp − minimum temp); bio3 = isothermality (bio2/bio7) (*100); bio4 = temperature seasonality (standard deviation *100); bio5 = maximum temperature of warmest month; bio6 = minimum temperature of coldest month; bio7 = temperature annual range (bio5‐bio6); bio8 = mean temperature of wettest quarter; bio9 = mean temperature of driest quarter; bio10 = mean temperature of warmest quarter; bio11 = mean temperature of coldest quarter; bio12 = annual precipitation; bio13 = precipitation of wettest month; bio14 = precipitation of driest month; bio15 = precipitation seasonality (coefficient of variation); bio16 = precipitation of wettest quarter; bio17 = precipitation of driest quarter; bio18 = precipitation of warmest quarter; bio19 = precipitation of coldest quarter; bio20 = sand content (gravimetric).

The environmental data used in the models were selected through Pearson's correlation by SDMtools in the ArcGIS software and by applying biological criteria (Table 1; Supporting Information Tables S1 and S2).^43^, ^46^ This selection was made to avoid multicollinearity, that is, to ensure that only one variable from each set, highly correlated (*r* ≥ | 0.75 |), was included in the model.^3^ Therefore, seven environmental variables relevant to the species (e.g. temperature, precipitation and soil texture are important variables that influence survival and development of the species) were retained in the final models (Table 2).

**Table 2:** Environmental variables considered in the niche model for *Striacosta albicosta* and their average percentage contribution to the model; the values were calculated using 10 repeated runs. Statistics were calculated using all occurrences (*n* = 750)

Description (variable)	Variable value average (minimum − maximum)	Characteristics of the selected model	
		Percentage contribution	Permutation importance
Mean annual range in temperature (bio7; °C)	37.71 (22.8–47.6)	34.5	19.5
Annual mean temperature (bio1; °C)	7.68 (2.0–17.4)	31.8	57.7
Precipitation of the driest month (bio14; mm)	53.4 (3–94.0)	23.0	0.0
Mean diurnal range in temperature (bio2; °C)	10.31 (8.1–18.6)	4.9	0.4
Precipitation seasonality (CV) (bio15)	20.3 (7.9–98.7)	3.3	4.7
Sand content (gravimetric) (bio20; %)	43.3 (5.8–71.2)	1.5	0.2
Mean annual precipitation (bio12; mm)	941.9 (248.0–1320.0)	1.1	17.4
Isothermality (bio3)	27.38 (22.3–68.3)	*	*
Temperature seasonality (SD × 100) (bio4)	1003.97 (174.5–1311.3)	*	*
Maximum temperature of the warmest month (bio5; °C)	26.75 (22.5–34.3)	*	*
Minimum temperature of the coldest month (bio6; °C)	−10,96 (−22.3–3.8)	*	*
Mean temperature of the wettest quarter (bio8; °C)	15.02 (−4.5–23.3)	*	*
Mean temperature of the driest quarter (bio9; °C)	−2.85 (−12.97–19.89)	*	*
Mean temperature of the warmest quarter (bio10; °C)	19.65 (15.0–24.3)	*	*
Mean temperature of the coldest quarter (bio11; °C)	−4.99 (−14.2–13.1)	*	*
Precipitation of the wettest month (bio13; mm)	100.32 (31.0–177.0)	*	*
Precipitation of the wettest quarter (bio16; mm)	283.0 (87.0–457.0)	*	*
Precipitation of the driest quarter (bio17; mm)	178.2 (12–289.0)	*	*
Precipitation of the warmest quarter (bio18; mm)	261.97 (33–381.0)	*	*
Precipitation of the coldest quarter (bio19; mm)	194.75 (19–374)	*	*

* Variables that were not selected for the model. Source of the data: WorldClim (http://www.worldclim.org/bioclim).

### 2.3 Model development and validation

Models for the current distribution and potential future distribution of *S. albicosta* and its hosts *Z. mays* and *P. vulgaris* were determined using the correlative model based on maximum entropy approach, using the MaxEnt software (v3.3.3k).^47^ MaxEnt was selected for its capability to generate accurate projections even with limited sample sizes and the requirement for only presence data of the pest and its hosts.^2^, ^6^ This software establishes relationships between species occurrence points and environmental variables to identify the potentially suitable areas for the species.^47^ The output of MaxEnt is a suitability index that ranges from 0 to 1, where 0 represents the areas that are unsuitable for the survival of the species and 1 represents areas that are highly suitable.^3^, ^6^ To generate global‐scale models, it is recommended to use 50 000 randomly selected background points from the current occurrence areas for each species. This number is used because it is appropriate for work on a global scale. Additionally, a sampling bias was generated for the species through the kernel density estimate in SDMToolbox, to compensate for the effect of the sampling intensity and the potential sampling tendency.^46^

In order to develop the models for *S. albicosta*, *Z. mays* and *P. vulgaris*, we adjusted different settings in the MaxEnt program to obtain different combinations of the regularization multiplier (RM) and resource classes.^48^ RMs are used to select the most influential resources in the model while controlling model complexity and overfitting. RM values <1 are restrictive and inadequate for global distribution predictions and values >1 generate simpler models with a broader potential distribution.^47^ Therefore, we explore combinations of resource classes including linear [L], quadratic [Q], product [P], threshold [T] and hinge [H], paired with RM values of 1.0, 1.5 and 2.0 to identify the optimal model for each species (Tables 3, S3 and S4).

**Table 3:** Summary of performance statistics of *Striacosta albicosta* MaxEnt models using the environmental variables bio1, bio2, bio7, bio12, bio14, bio15 and bio20. The best model is presented in bold

Model Rank	MaxEnt settings		Test AUC_cv_ (±SD)	OR		AICc	BIC
	Features	RM		0%	10%		
**1**	**LQP**	**1**	**0.9738 ± 0.0040**	**0.0020**	**0.1039**	**7579.18**	**7638.18**
2	LQH	2	0.9790 ± 0.0031	0.0021	0.1040	7582.03	7639.32
3	LQPH	1.5	0.9796 ± 0.0030	0.0021	0.1082	7585.04	7643.18
4	LQPH	1	0.9799 ± 0.0030	0.0021	0.1122	7586.13	7645.43
5	LQP	2	0.9735 ± 0.0039	0.0021	0.1123	7588.22	7647.24
6	LQH	1.5	0.9788 ± 0.0032	0.0021	0.1125	7591.45	7649.03
7	LQPTH	1.5	0.9775 ± 0.0036	0.0021	0.1143	7594.19	7649.92
8	LQPT	2	0.9760 ± 0.0037	0.0021	0.1163	7598.37	7651.43
9	LQPT	1	0.9773 ± 0.0043	0.0021	0.1268	7599.17	7652.67

*Note*: For variables' full names, see Table 1. L, Q, P, T and H, linear, quadratic, product, threshold and hinge features, respectively; RM, regularization multiplier; SD, standard deviation; and OR, test omission rate. Test AUC_cv_ is the MaxEnt 10‐fold cross‐validation area under the ROC curve. AICc, Akaike information criterion; and BIC, Bayesian information criterion.

In order to eliminate extrapolations outside the environmental range, we selected MaxEnt's ‘fade‐by‐clamping’ option.^49^ We also used the ‘Jackknife’ method to estimate the predictive contribution of environmental variables, estimate their importance of permutation, and generate species‐response curves. These response curves allow for the analysis of the relationship between the probability of species presence and each environmental predictor.^47^ All response curves were evaluated based on biological logic to verify the significance of each environmental variable's effect on species occurrence probability.^2^ Variables that did not align with biological expectations were excluded from the analysis.

In order to compare the performance of the models, we calculated the Bayesian Information Criterion (BIC) using ENMTools for the selection of the best model.^50^ We chose this criterion because it has a higher penalty for the model complexity than the Akaike Information Criterion (AIC). Similar to AIC a smaller BIC value means a ‘better’ model.^4^ Additionally, to verify the performance characteristic of the models, a 10‐fold cross‐validation was performed using MaxEnt. This allows us to calculate the area under the receiver operating characteristic (ROC) curve (AUCcv)^51^ and the sensitivity of the test of omission rates (OR) of 0% and 10%.^4^, ^51^ AUCcv also was used to discriminate the presence of background data. AUCcv values classify model performance as follows: predictions worse than random (AUCcv <0.5), no better than random (AUCcv = 0.5), low performance (AUCcv <0.7), satisfactory to moderate performance (0.7 ≤ AUCcv <0.9) and high performance (AUCcv ≥0.9).^51^ Omission rates (OR) refer to the proportion of known occurrences of the species that are classified as absent by the model. The OR represents, in percentage (0% and 10%), the value of training for the model. An omission rate of 0% means that all known occurrences of the species are correctly predicted by the model. And an omission rate of 10% indicates that 10% of the known occurrences of the species are not predicted by the model–thus, they are classified as absent. Therefore, regarding the sensitivity of the test at 0% and 10%, the OR threshold of the training must be 0 and 0.10, respectively. Thus, poor performance of the model is indicated when the value exceeds the values of the expected rate.^42^

The Maximum Test Sensitivity Plus Specificity (MTSPS) threshold was selected to extract the projected distributions for each species. MTSPS was used because it is considered a simple and effective method for averaging probability and suitability categories, which accurately defines the suitability class and reduces false positives.^52^ We determined suitability classes for the hosts *Z. mays* and *P. vulgaris*, as well as for the pest *S. albicosta*, indicating which areas would be optimal for planting corn and dry beans and the associated risk levels for *S. albicosta* (unsuitable: 0‐MTSPS; suitable: MTSPS‐0.5; and high suitability: 0.5–1.0).

### 2.4 Future projections

The potential distributions of the pest *S. albicosta* and its hosts *Z. mays* and *P. vulgaris*, under the climate change scenario, were modeled for the years 2050 and 2070. For this purpose, we used the global climate model (GCM) MIROC5 under representative concentration pathway 4.5 (RCP4.5). MIROC5 is one of the models used in the Intergovernmental Panel on Climate Change (IPCC) Fifth Assessment Report (AR5) and associated cycle of the fifth phase of CMIP5 (http://www.ipcc.ch/report/ar5/wg1/).^53^ The model considers several factors, including the impact of emissions of greenhouse gases, aerosols, solar irradiance, ozone and other pollutants that contribute to global temperature increases.^54^ RCP4.5 is an intermediate scenario used to project climate change effects. This scenario predicts a temperature increase ranging between 1.1 and 2.6°C by the end of the 21st Century.^55^ We use this scenario because it is likely to occur^56^ and for predicting changes in global rainfall standard. RCP4.5 was developed by the Global Change Assessment Model (GCAM) team at the Joint Global Change Research Institute (JGCRI) of the Pacific Northwest National Laboratory (http://www.globalchange.umd.edu/models/gcam/).^2^

## 3 RESULTS

**Figure 1:**
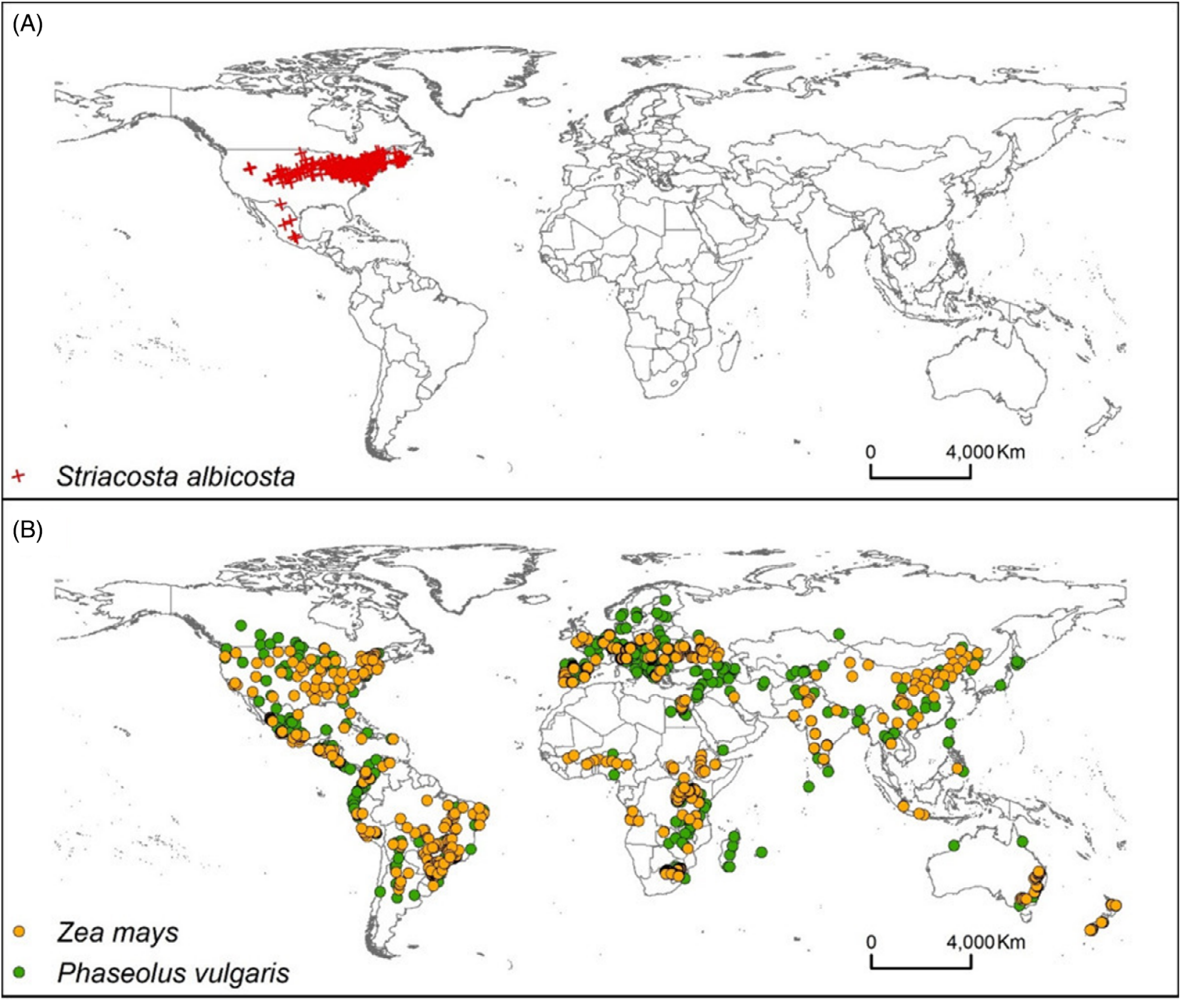
Distribution points of (A) *Striacosta albicosta* and (B) *Zea mays* (orange dots) and *Phaseolus vulgaris* (green dots).

Distribution points used in the present study for *S. albicosta* and its hosts *Z. mays* and *P. vulgaris* are shown in Fig. 1. The environmental variables that most contributed to the distribution of *S. albicosta* were the mean annual range in temperature, annual mean temperature, precipitation of the driest month, mean diurnal range in temperature, precipitation seasonality, sand content (gravimetric) at 5‐cm soil depth, and mean annual precipitation (Table 2). Among the variables, the mean annual range in temperature was the variable that presented the highest percentage contribution (34.5%), followed by annual mean temperature (31.8%) and precipitation of the driest month (23%) (Table 2).

The climatic variables that contributed to the distribution of *Z. mays* and *P. vulgaris* were annual mean temperature, mean annual precipitation, sand content (gravimetric), mean diurnal range in temperature, temperature annual range, precipitation seasonality and precipitation of the driest month. The order of importance of these variables varied between the two species (Tables S1 and S2).

All of the performance statistics of the *S. albicosta* MaxEnt models are provided in Table 3. The average BIC values ranged from 7638.18 to 7652.67 and AICc values ranged from 7579.18 to 7599.17. Combinations of RMs and types of resource classes were tested to select the best model for *S. albicosta* and its hosts *Z. mays* and *P. vulgaris* (Tables 3, S3 and S4). All models performed well, with low omission rates (OR) at 0% and 10% and AUCcv values greater than 0.7 (Tables 3, S3 and S4). The AUCcv values ranged from 0.9735 to 0.9799 for *S. albicosta* (Table 3), 0.8623 to 0.8819 for *Z. mays* (Table S3), and 0.8491 to 0.8551 for *P. vulgaris* (Table S4). The ORs for *S. albicosta* that were lowest at 0% and 10% were 0.0020 and 0.1039 (Table 3). The lowest ORs at 0% and 10% for the hosts were 0.0015 and 0.1096 for *Z. mays*, and 0.0055 and 0.1023 for *P. vulgaris* (Tables S3 and S4). Thus, the best model for *S. albicosta* included seven environmental parameters, linear, quadratic and product characteristics (LQP), and regularization multiplier (RM) = 1, resulting in the lowest ORs at 0% and 10% (Table 3). In the same way, for the hosts, the best model had seven environmental parameters, the characteristics were linear and hinge (LH) with RM = 1 for *Z. mays*, and for *P. vulgaris* the characteristics were linear, quadratic, product and hinge (LQPH) and RM = 2 (Tables S3 and S4).

**Figure 2:**
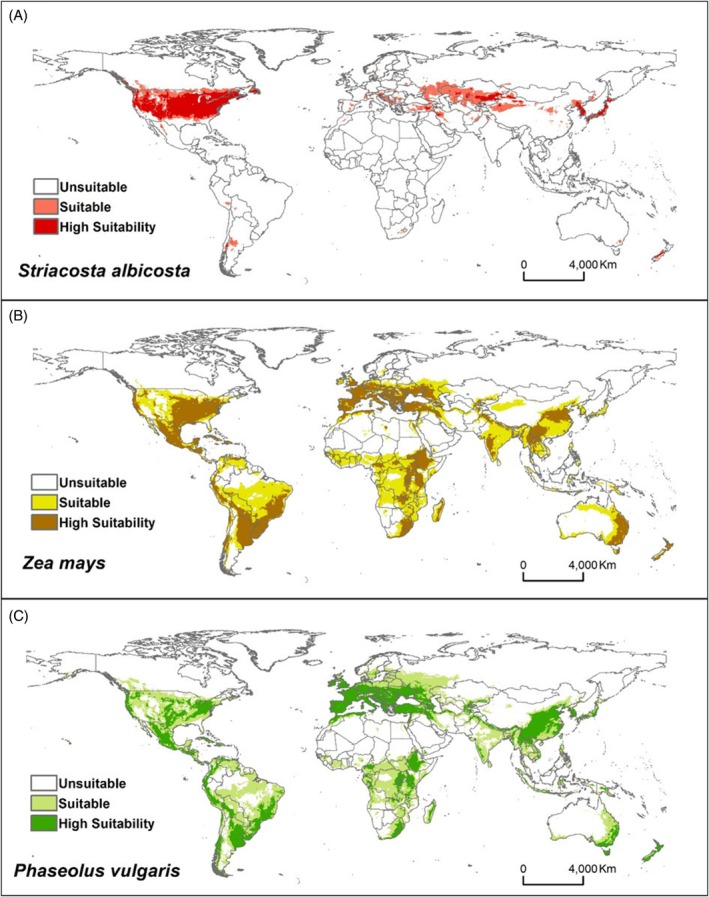
Habitat suitability under current climate conditions using the MaxEnt model for (A) *Striacosta albicosta*, (B) *Zea mays* and (C) *Phaseolus vulgaris*.

The known occurrence areas of *S. albicosta*, *Z. mays* and *P. vulgaris* coincided with those projected by the MaxEnt model, suggesting good predictive performance (Figs 1 and 2). According to the model projections, 11.41% of the area was classified as suitable, whereas 8.81% was identified as highly suitable for *S. albicosta*. Most areas of occurrence of the pest species *S. albicosta* have been projected as highly adequate, mainly in the United States (Figs S1 and S2). Currently, there are records of the pest in 23 states of the United States. In addition to these current regions of occurrence, an additional 24 more US states, along with areas in South America, Europe, Asia, Oceania and two countries in Africa (Morocco and South Africa), are identified as suitable or highly suitable for the potential introduction and establishment of this pest (Figs 2, S3 and S4). Although several countries around the world have suitable areas for growing corn and dry beans, the majority of these regions are not suitable for the establishment of *S. albicosta* (Figs 2, S3 and S4).

**Figure 3:**
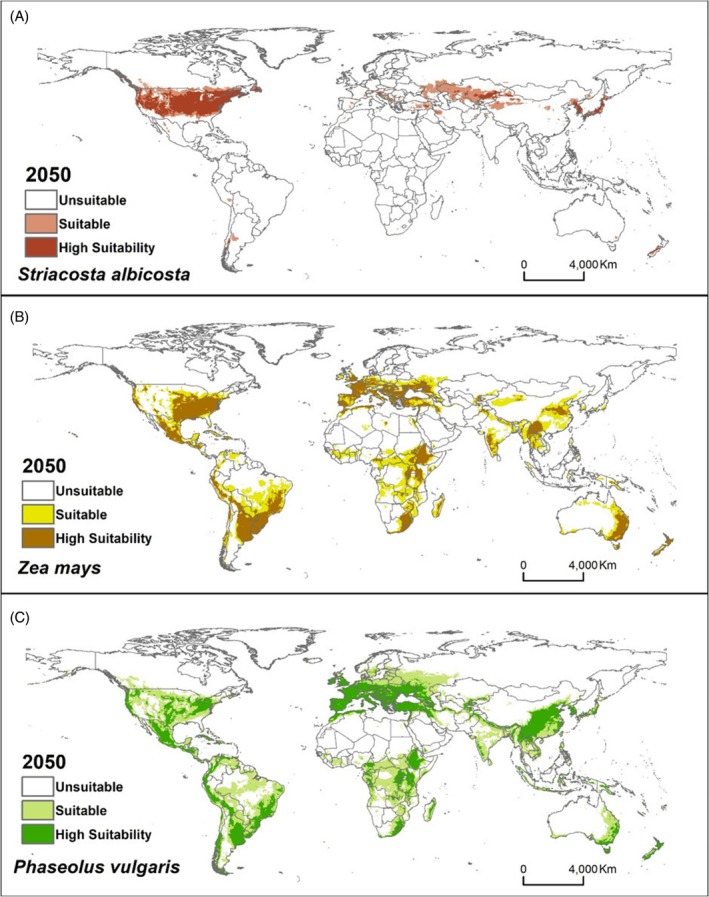
Habitat suitability under future climatic (2050) conditions for (A) *Striacosta albicosta*, (B) *Zea mays* and (C) *Phaseolus vulgaris* using MaxEnt models running the MIROC5 (GCM) under the RCP 4.5 scenario.

**Figure 4:**
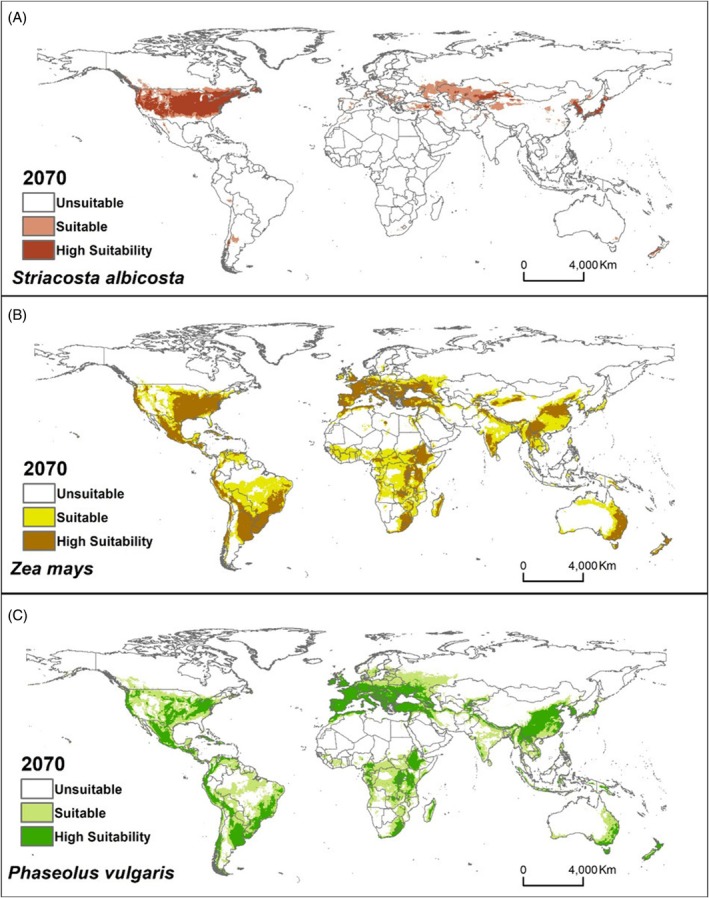
Habitat suitability under future climatic (2070) conditions for (A) *Striacosta albicosta*, (B) *Zea mays* and (C) *Phaseolus vulgaris* using MaxEnt models running the MIROC5 (GCM) under the RCP 4.5 scenario.

In future projections for the years 2050 and 2070 under the climate change scenario RCP4.5, notable reductions in suitable areas are predicted for *Z. mays*, particularly in regions of South America, Central Africa, Oceania and Asia [Figs 3(B), 4(B) and S3]. Conversely, for *S. albicosta* and its other host, *P. vulgaris*, there may be slight reductions in the extent of suitable areas compared to present conditions; however, these changes are minimal and cannot be seen on the maps, indicating relatively stable distributions for both species [Figs 3(A),(C), 4(A),(C) and S4]. Although this study focused on RCP 4.5 as a moderate and policy‐relevant climate scenario, it is important to note that more severe scenarios (e.g. RCP 6.0 or 8.5) and more up‐to‐date scenarios (e.g. SSPs) could result in highly vulnerable regions, such as West Africa. Future work should explore these scenarios to fully capture the potential extent of the pest's spread under varying climate futures.

**Figure 5:**
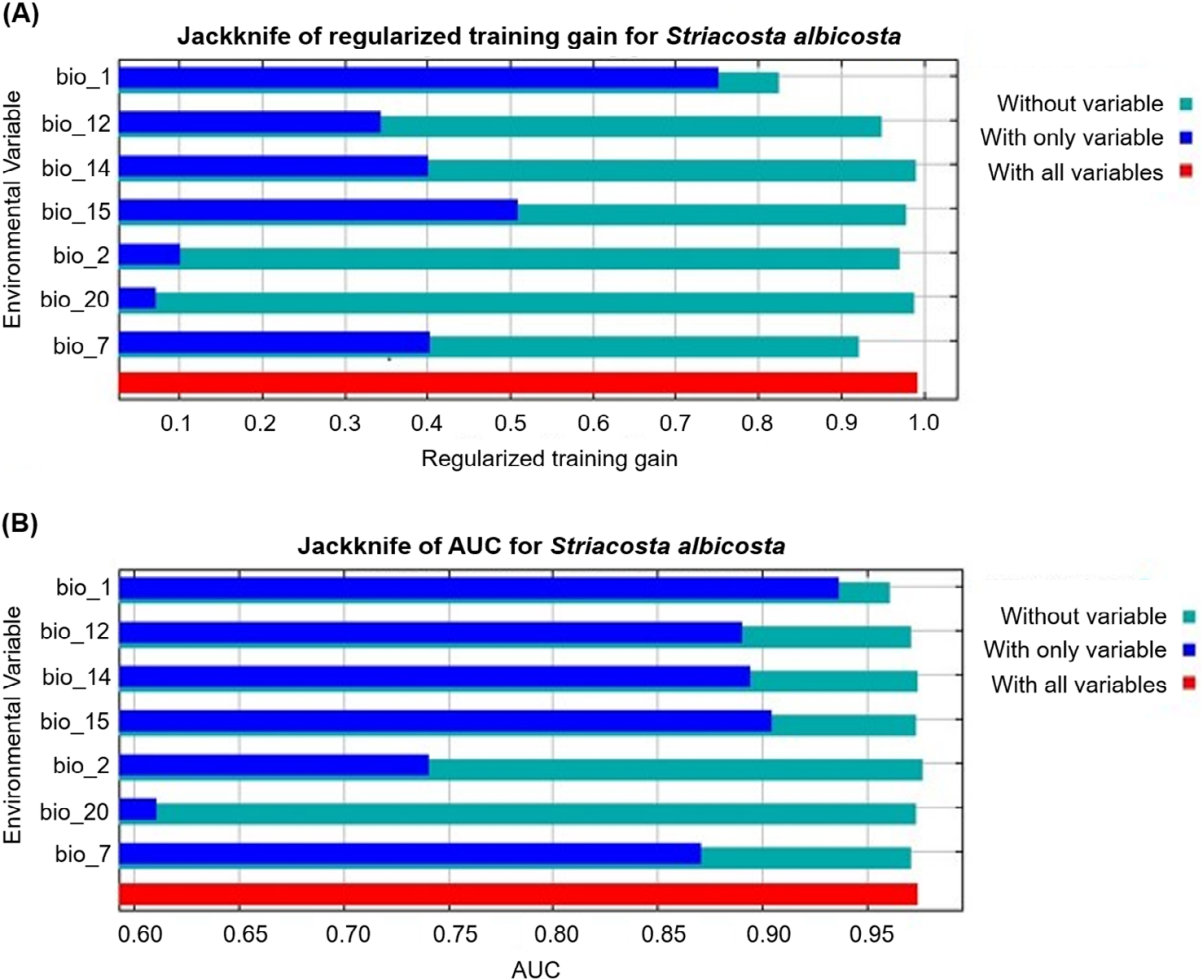
Relative importance of environmental variables based on the JackKnife test of (A) regularized training gain and (B) AUC in the *Striacosta albicosta* model.

**Figure 6:**
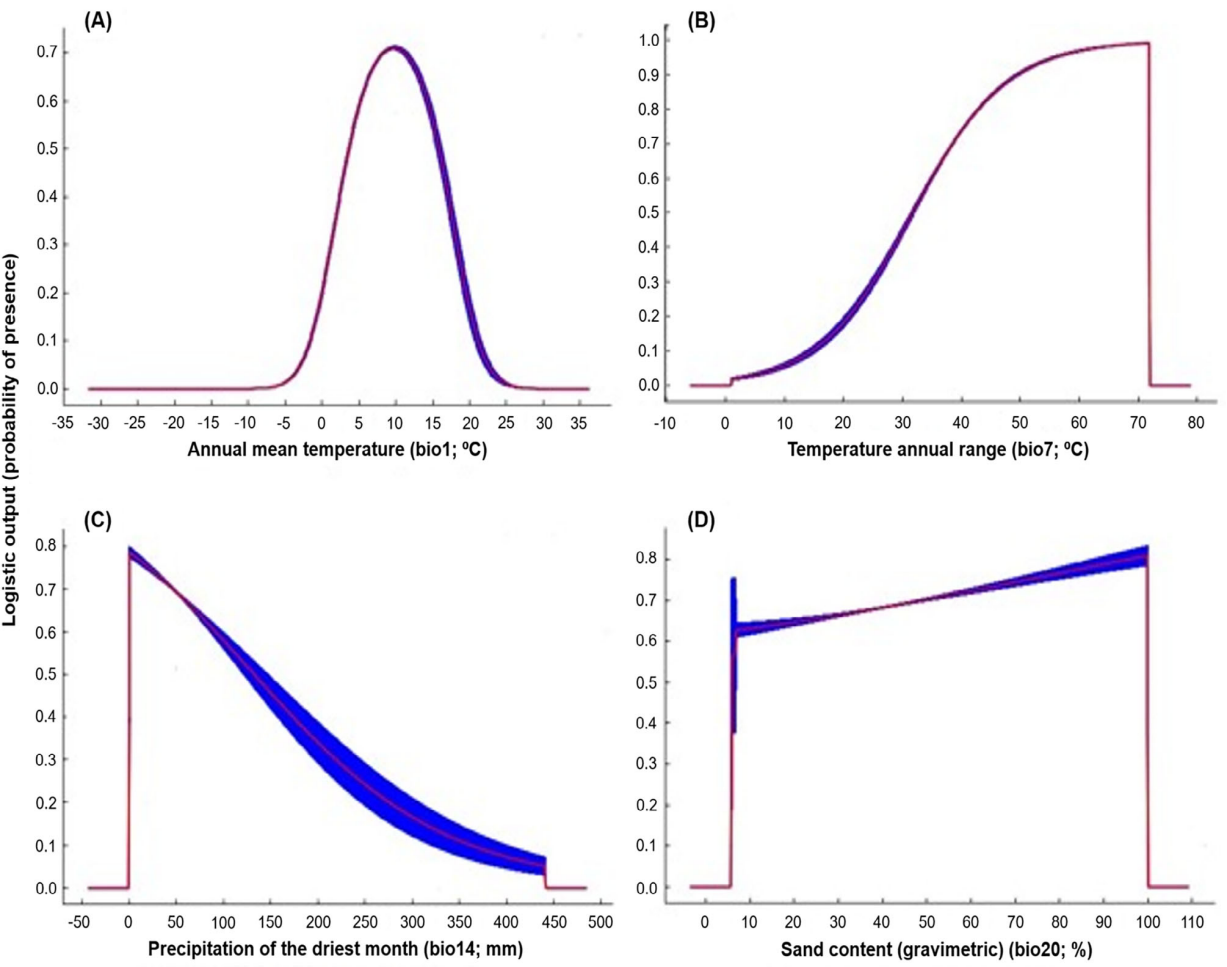
Response curves of the best predictors of *Striacosta albicosta* in the best model: (A) annual mean temperature (bio1; °C), (B) temperature annual range (bio7; °C), (C) precipitation of the driest month (bio14; mm) and (D) sand content (gravimetric) (bio20; %).

The ‘Jackknife’ test showed that the average annual temperature had the greatest impact on the model for all species (*S. albicosta*, *Z. mays* and *P. vulgaris*), with the highest gain in regularized training and AUC (Figs 5, S5 and S6). The relationships between the predicted probabilities of species presence and environmental parameters are represented in Figs 6, S7 and S8. On the one hand, the greatest probability for the presence of *S. albicosta* occurs in areas with average annual temperatures of ≈10°C, declining drastically with increasing or decreasing average annual temperature, with no prediction of occurrence in temperatures below −5 °C or above 25 °C (Fig. 6). On the other hand, the greatest probability of the presence of host species is in places with average annual temperatures of 8–19 °C for *Z. mays* (Fig. S7) and of 10 °C for *P. vulgaris* (Fig. S8). Regarding the precipitation of the driest month variable, the probability of the presence of *S. albicosta* is higher in areas of low precipitation. With increasing precipitation, the probability of the presence of this species decreases (Fig. 6).

## 4 DISCUSSION

This is the first study conducted to estimate suitable areas for the western bean cutworm, *S. albicosta*, globally. As this insect has become an important pest of corn and dry beans in the United States and Canada, knowing the suitable areas for its introduction and establishment under current and climate change scenarios is fundamental for effective pest control planning.

The models generated in this study exceeded randomness, demonstrated through AUCcv. When a model overcomes randomness, it indicates that the projected data are likely to be consistent with the actual species data.^3^, ^51^ Thus, the results of these models, along with their spatial overlays on the global distribution of host species, and the areas projected as suitable for the development of *S. albicosta* are statistically and biologically significant.

Biotic factors (such as host plants) and abiotic factors (such as temperature, precipitation and soil) contribute to insect development and expansion.^15^, ^57^ The variables related to temperature (mean annual range in temperature, annual mean temperature and mean diurnal range in temperature) had the strongest influence on the projected areas considered suitable for the development of western bean cutworm. In total, these variables contributed 71.2% of the proposed model. In addition to variables related to temperature, precipitation and sand content also proved significant (Table 2).

Several authors have reported the effect of temperature on the occurrence and development of *S. albicosta* under laboratory and field conditions. Both the immature and adult stages of western bean cutworm are temperature‐sensitive.^15^ Under controlled conditions with an average temperature of 26 °C, the complete life cycle can vary from 95 to 150 days.^15^ Konopka and McNeil^57^ report that variations between diurnal and nocturnal temperatures result in a difference in the sexual maturation time of *S. albicosta*; when the diurnal temperature variation is high (10 °C or more), females reach sexual maturity more quickly. A study carried out by Hanson *et al*.^58^ demonstrates that the flight time of the pest species *S. albicosta* is significantly affected by the average temperature variation. Thus, the significance of temperature‐related variables in the literature corroborates the accuracy of our estimates of suitable areas for *S. albicosta*.

Studies on pest distribution modeling are quite useful, but it is important to remember that they also have some limitations, such as representativeness of occurrence data, host availability and climate scenarios, which compromise the accuracy of the models. Additionally, the factors that compromise the physiology of the species under study are not always known, so they often cannot be included in the set of predictors.^59^, ^60^

Thus, constructing prediction models for species occurrence using locations where the species is currently found is essential for validating, forecasting and refining the variables that significantly influence the establishment and development of the species under study.^6^, ^56^

Part of the life cycle of *S. albicosta* occurs in the soil. After completing its larval stage feeding on its host plants, the caterpillar then develops into a prepupa stage that burrows into the soil, and builds individual chambers where they overwinter and remain throughout the pupal stage, before emerging as an adult.^61^ Therefore, soil texture may influence both the development and distribution of the species. Soils with higher sand content can facilitate larval entry and prepupal chamber formation, as they offer less resistance to digging. They may also provide better protection against low temperatures during the winter by allowing caterpillars to burrow deeper. By contrast, very compact soils can hinder adult emergence.^12^, ^61^, ^62^ Thus, considering this environmental variable is important in modeling studies based on species occurrence and ecological niche, given that the western bean cutworm's ability to overwinter depends on soil type.^61^

In the current climate scenario, only a few areas in temperate regions were projected as suitable for the pest species *S. albicosta*. In addition to the known occurrence in North America, small parts of South America, Europe, Asia, Oceania and Africa are possibly suitable for *S. albicosta*. Temperate regions are cooler and have four well‐defined seasons, resulting in high seasonal and daily temperature variations.^63^ Our study confirms that the climate variables, especially annual temperature range and mean annual temperature, were the main predictors for the distribution of *S. albicosta*, and so received the highest weights in the model.

The limitation on the projection of suitable areas for *S. albicosta* is therefore not primarily caused by soil conditions, as it is dominated by climatic variables. Even in regions with suitable soils, climate is likely to influence the projection of suitable areas for the pest on a global scale. This insect is endemic to temperate regions and possibly depends on well‐defined seasonal temperature conditions throughout the seasons for its development and life cycle.^61^ Thus, regions with low thermal variation and consistently high temperatures^64^ may limit important stages of the insect's development. Although high temperatures generally favor the development of many insects, the lack of specific data on the thermal limits on the survival rate of *S. albicosta* ^61^, ^62^ may hinder more accurate projections. These factors may explain the small extent of suitable areas projected for the species globally.

The current distribution model of the host species *Z. mays* and *P. vulgaris* demonstrates that tropical, as well as temperate, regions of the six continents (North and South America, Africa, Europe, Asia and Oceania), are suitable for planting these species. This is possibly a consequence of domestication and the diversity of varieties adapted to the environmental conditions in these regions, from tropical to cold temperate.^65^

In this study, we also generate future projections for the pest and its host species. For *S. albicosta* and *P ulgaris*, the areas projected as suitable will remain the same as in the current climate scenario. However, the areas suitable for *Z. mays* will decrease in some countries within the tropical zones of the Americas, Africa and Asia. It is estimated that, due to climate change, very high temperatures will occur more frequently and will be harmful to corn phenology. It is reported that higher temperatures during the corn growing season will reduce crop yield in several regions of the world,^66^ which corroborates the projections of this work.

In the overlap projection (Figs S3 and S4), we demonstrate areas with optimal conditions for the pest *S. albicosta* as well as its hosts *Z. mays* and *P. vulgaris*. These areas potentially suitable for both *S. albicosta* and *Z. mays* species are in North American countries, Chile, Argentina, and some European and Asian countries. The areas potentially suitable for *S. albicosta* and *P. vulgar*is are those described previously for maize, with the addition of New Zealand.

Although the models estimate that some countries in the tropical zone have suitable areas for *S. albicosta* and its hosts, this pest is currently found only in North American countries: the United States, Canada and Mexico.^11^, ^12^, ^15^ The absence of this pest in other countries with projected suitable areas is likely to be a result of the difficulty of dispersal, usually imposed by natural barriers such as seas and deserts.^43^, ^67^

In both the current and future climate scenario projections, 47 U.S. states and five Canadian provinces have been identified as potentially suitable areas for this pest. This evidence highlights the potential need to implement strategies such as inspection and quarantine barriers to prevent entry and establishment in areas still free of *S. albicosta* in the U.S., because this is the world's main producer of corn.^41^ Currently, there are reports of the pest in 24 U.S. states, and it may disperse to areas determined as suitable in the other 23 currently unoccupied states. Therefore, our results will help in actions to alert government institutions to use management strategies and promote phytosanitary biosecurity.

## 5 CONCLUSION

Our study evaluated the areas suitable for the distribution of *S. albicosta*, an important pest of corn and dry beans, given the current scenario and under the effect of climate change. The models in this study were based on climatic parameters and an edaphic characteristic (soil sand content). The available global data on climatic and soil sand content, as well as field occurrence data of the pest species *S. albicosta* and its hosts *Z. mays* and *P. vulgaris* were used. The designed models showed accordance with the current distribution of the insect and its hosts. Among the climatic factors, the variables related to temperature and precipitation were the predictors that most influenced the distribution of *S. albicosta*. Currently, the occurrence of *S. albicosta* is restricted to North America, although our results indicated areas with climatic suitability for its establishment in other countries in South America, Africa, Europe, Asia and Oceania, and for its expansion in other U.S. states. As a result of the damage caused by this pest to corn and dry beans, the United States, as well as other countries that have presented suitable climatic conditions for this pest and its hosts, should consider defining phytosanitary measures such as the inspection of plants, plant parts and byproducts in transit and the monitoring of production areas. Our study can serve as a guide for government agencies to implement strategies to prevent the incursion and establishment of *S. albicosta* in areas still free of this pest.

## FUNDING INFORMATION

This study was financed in part by the Programa Nacional de Cooperação Acadêmica na Amazônia (PROCAD AMAZÔNIA—Process: 88881.357579/2019‐01) of the Coordenação de Aperfeiçoamento de Pessoal de Nível Superior—CAPES, Brazil. The Agroecosystems Entomology Lab at University of Nebraska‐Lincoln, West Central Research, Extension and Education Center was supported by the Nebraska Agricultural Experiment Station with funding from the Hatch Multistate Research capacity funding program (Accession no. 1006556) from the USDA National Institute of Food and Agriculture.

## CONFLICT OF INTEREST

The authors declare that they have no conflict of interest.

## AUTHOR CONTRIBUTIONS

Conceptualization, P.S.P. and J.A.P.; data curation, P.S.P., J.A.P., K.A.S.B., R.S.R., M.C.P. and R.A.S.; formal analysis, P.S.P. and R.S.R.; writing‐original draft preparation, P.S.P. and J.A.P.; writing‐review and editing, R.S.R., K.A.S.B., M.C.P., and R.A.S.; funding acquisition, M.C.P., and R.A.S. All authors have read and agreed to the published version of the manuscript.

## Supporting information


**Table S1.** Environmental variables considered in the niche model for *Zea mays* and their average percent contribution to the model; the values were calculated using 10 repeated runs. Statistics were calculated using all occurrences (*n* = 796).
**Table S2.** Environmental variables considered in the niche model for *Phaseolus vulgaris* and their average percentage contribution to the model; the values were calculated using 10 repeated runs. Statistics were calculated using all occurrences (*n* = 365).
**Table S3.** Summary of performance statistics of *Zea mays* MaxEnt models. The best model is highlighted in bold.
**Table S4.** Summary of performance statistics of *Phaseolus vulgaris* MaxEnt models. The best model is highlighted in bold.
**Figure S1.** Habitat suitability in the USA under current and future climatic conditions in optimal areas for *Zea mays* cultivation with three suitability levels of *Striacosta albicosta*. Maps (A) current time, (B) 2050 and (C) 2070.
**Figure S2.** Habitat suitability in the USA under current and future climatic conditions in optimal areas for *Phaseolus vulgaris* cultivation with three suitability levels of *Striacosta albicosta*. Maps (A) current time, (B) 2050 and (C) 2070.
**Figure S3.** Habitat suitability under current and future climatic conditions in optimal areas for *Zea mays* cultivation with three suitability levels of *Striacosta albicosta*. Maps (A) current time, (B) 2050 and (C) 2070.
**Figure S4.** Habitat suitability under current and future climatic conditions in optimal areas for *Phaseolus vulgaris* cultivation with three suitability levels of *Striacosta albicosta*. Maps (A) current time, (B) 2050 and (C) 2070.
**Figure S5.** Relative importance of environmental variables based on the JackKnife test (A) regularized training gain and (B) AUC in the *Zea mays* model.
**Figure S6.** Relative importance of environmental variables based on the JackKnife test (A) regularized training gain and (B) AUC in the *Phaseolus vulgaris* model.
**Figure S7.** Response curves of the best predictors of *Zea mays* in the best model. (A) annual mean temperature (bio1; °C), (B) temperature annual range (bio7; °C), (C) Precipitation of the driest month (bio14; mm) and (D) sand content (gravimetric) (bio20; %).
**Figure S8.** Response curves of the best predictors of *Phaseolus vulgaris* in the best model. (A) annual mean temperature (bio1; °C), (B) temperature annual range (bio7; °C), (C) Precipitation of the driest month (bio14; mm) and (D) sand content (gravimetric) (bio20; %).

## Data Availability

The data that support the findings of this study are available from the corresponding author upon reasonable request.
