# Supplementary material for: Modeling of suitable geographic areas for Striacosta albicosta in corn and dry bean crops under climate change scenarios
Source: Pest Manag Sci. 2025 Sep 29;82(1):1121–33. doi: 10.1002/ps.70270 (PMC12713701; doi:10.1002/ps.70270)
Supplement: Supplementary file 1 — Table S1. Environmental variables considered in the niche model for Zea mays and their average percent contribution to the model; the values were calculated using 10 repeated runs. Statistics were calculated using all occurrences (n = 796). Table S2. Environmental variables considered in the niche model for Phaseolus vulgaris and their average percentage contribution to the model; the values were calculated using 10 repeated runs. Statistics were calculated using all occurrences (n = 365). Table S3. Summary of performance statistics of Zea mays MaxEnt models. The best model is highlighted in bold. Table S4. Summary of performance statistics of Phaseolus vulgaris MaxEnt models. The best model is highlighted in bold. Figure S1. Habitat suitability in the USA under current and future climatic conditions in optimal areas for Zea mays cultivation with three suitability levels of Striacosta albicosta. Maps (A) current time, (B) 2050 and (C) 2070. Figure S2. Habitat suitability in the USA under current and future climatic conditions in optimal areas for Phaseolus vulgaris cultivation with three suitability levels of Striacosta albicosta. Maps (A) current time, (B) 2050 and (C) 2070. Figure S3. Habitat suitability under current and future climatic conditions in optimal areas for Zea mays cultivation with three suitability levels of Striacosta albicosta. Maps (A) current time, (B) 2050 and (C) 2070. Figure S4. Habitat suitability under current and future climatic conditions in optimal areas for Phaseolus vulgaris cultivation with three suitability levels of Striacosta albicosta. Maps (A) current time, (B) 2050 and (C) 2070. Figure S5. Relative importance of environmental variables based on the JackKnife test (A) regularized training gain and (B) AUC in the Zea mays model. Figure S6. Relative importance of environmental variables based on the JackKnife test (A) regularized training gain and (B) AUC in the Phaseolus vulgaris model. Figure S7. Response curves of the best predic [file PS-82-1121-s001.docx]

**Supporting Information**

**Modeling of suitable geographic areas for *Striacosta albicosta* in corn and dry bean crops under climate change scenarios**

Poliana S Pereira^a*^, Julie A Peterson^b^, Rodrigo S Ramos^c^, Katharine A Swoboda Bhattarai^b^, Marcelo C Picanço^c^, Renato A Sarmento^a^

^a^ Postgraduate Program in Plant Production, Federal University of Tocantins, Gurupi, TO, Brazil

^b^ Departament of Entomology, University of Nebraska-Lincoln, West Central Research, Extension & Education Center, North Platte, NE, United States

^c^ Departament of Entomology, Federal University of Viçosa, Viçosa, MG, Brazil

^*^ Correspondence to: PS Pereira, Postgraduate Program in Plant Production, Federal University of Tocantins, Gurupi 77410-530, TO, Brazil. E-mail: [poliana_silvestre@yahoo.com.br](mailto:poliana_silvestre@yahoo.com.br)

**This file includes:**

Tables S1 to S4

Figures S1 to S8

**Other Supporting Information for this manuscript include the following:** spreadsheets with coordinate data (latitude and longitude) of occurrence of the pest, *Striacosta albicosta*, and its hosts, *Zea mays* and *Phaseolus vulgaris*.

**Table S1.** Environmental variables considered in the niche model for *Zea mays* and their average percent contribution to the model; the values were calculated using 10 repeated runs. Statistics were calculated using all occurrences (n = 796).

| Description (variable) | Variable value average (minimum - maximum) | Characteristics of the selected model | |
| --- | --- | --- | --- |
|  |  | Percent Contribution | Permutation Importance |
| Annual mean temperature (bio1; °C) | 16.5 (1.7 - 28.9) | 63.1 | 55.2 |
| Mean annual precipitation (bio12; mm) | 951.0 (3.0 - 3988.0) | 21.4 | 13.2 |
| Sand content (gravimetric) (bio20; %) | 42.6 (10.1 - 83.1) | 8.1 | 14.4 |
| Mean diurnal range in temperature (bio2; °C) | 11.7 (6.1 - 18.2) | 4.2 | 8.1 |
| Temperature annual range (bio7; °C) | 25.1 (9.7 - 53.7) | 2.6 | 6.0 |
| Precipitation seasonality (CV) (bio15) | 55.6 (6.4 - 149.1) | 0.4 | 2.1 |
| Precipitation of the driest month (bio14; mm) | 28.05 (0 - 142.0) | 0.2 | 0.9 |
| Isothermality (bio3) | 51.8 (20.9 - 92.8) | * | * |
| Temperature seasonality (SD × 100) (bio4) | 477.1 (10.7 - 1566.3) | * | * |
| Maximum temperature of the warmest month (bio5; °C) | 29.1 (18.2 - 44.5) | * | * |
| Minimum temperature of the coldest month (bio6; °C) | 3.9 (-26.6 - 21.9) | * | * |
| Mean temperature of the wettest quarter (bio8; °C) | 19.1 (0.3 - 33.4) | * | * |
| Mean temperature of the driest quarter (bio9; °C) | 13.5 (-17.8 - 35.0) | * | * |
| Mean temperature of the warmest quarter (bio10; °C) | 22.2 (10.7 - 35.0) | * | * |
| Mean temperature of the coldest quarter (bio11; °C) | 10.5 (-17.8 - 27.1) | * | * |
| Precipitation of the wettest month (bio13; mm) | 152.9 (1.0 - 1087.0) | * | * |
| Precipitation of the wettest quarter (bio16; mm) | 403.4 (2.0 - 2818.0) | * | * |
| Precipitation of the driest quarter (bio17; mm) | 100.5 (0 - 476.0) | * | * |
| Precipitation of the warmest quarter (bio18; mm) | 278.1 (0 - 1279.0) | * | * |
| Precipitation of the coldest quarter (bio19; mm) | 171.4 (0 - 999) | * | * |

Variables that were used in the final model.

*Variables that were not selected for the model.

Source of the data: WorldClim (http://www.worldclim.org/bioclim).

**Table S2.** Environmental variables considered in the niche model for *Phaseolus vulgaris* and their average percentage contribution to the model; the values were calculated using 10 repeated runs. Statistics were calculated using all occurrences (n = 365).

| Description (variable) | Variable value average (minimum - maximum) | Characteristics of the selected model | |
| --- | --- | --- | --- |
|  |  | Percent Contribution | Permutation Importance |
| Annual mean temperature (bio1; °C) | 14.94 (0.6 - 28.1) | 63.5 | 58.1 |
| Mean annual precipitation (bio12; mm) | 945.3 (1.0 - 3951.0) | 22.0 | 8.1 |
| Sand content (gravimetric) (bio20; %) | 42.7 (19.3 - 88.7) | 7.9 | 13.4 |
| Temperature annual range (bio7; °C) | 25.7 (5.9 - 55.5) | 3.4 | 16.2 |
| Precipitation of the driest month (bio14; mm) | 26.6 (0 - 133.0) | 1.9 | 1.8 |
| Mean diurnal range in temperature (bio2; °C) | 10.93 (4.3 - 19.0) | 1.1 | 2.2 |
| Precipitation seasonality (CV) (bio15) | 56.1 (9.1 - 142.9) | 0.2 | 0.1 |
| Isothermality (bio3) | 49.1 (20.1 - 92.0) | * | * |
| Temperature seasonality (SD × 100) (bio4) | 532.34 (13.6 - 1615.1) | * | * |
| Maximum temperature of the warmest month (bio5; °C) | 28.0 (13.8 - 41.3) | * | * |
| Minimum temperature of the coldest month (bio6; °C) | 2.4 (-29.3 - 25.4) | * | * |
| Mean temperature of the wettest quarter (bio8; °C) | 17.3 (0.2 - 31.1) | * | * |
| Mean temperature of the driest quarter (bio9; °C) | 12.7 (-20.5 - 30.4) | * | * |
| Mean temperature of the warmest quarter (bio10; °C) | 21.3 (8.9 - 32.5) | * | * |
| Mean temperature of the coldest quarter (bio11; °C) | 8.3 (-20.5 - 27.7) | * | * |
| Precipitation of the wettest month (bio13; mm) | 152.5 (1.0 - 620.0) | * | * |
| Precipitation of the wettest quarter (bio16; mm) | 401.9 (1.0 - 1685.0) | * | * |
| Precipitation of the driest quarter (bio17; mm) | 95.7 (0 - 446.0) | * | * |
| Precipitation of the warmest quarter (bio18; mm) | 270.8 (0 - 1487.0) | * | * |
| Precipitation of the coldest quarter (bio19; mm) | 177.1 (0 - 1029) | * | * |

Variables that were used in the final model.

*Variables that were not selected for the model.

Source of the data: WorldClim (http://www.worldclim.org/bioclim).

**Table S3.** Summary of performance statistics of *Zea mays* MaxEnt models. The best model is highlighted in bold.

| **ModelRank** | **Variables** | **MaxEnt settings** | | **Test AUC_cv_**  **(±SD)** | **OR** | |
| --- | --- | --- | --- | --- | --- | --- |
|  |  | **Features** | **RM** |  | **0%** | **10%** |
| **1** | **bio1, bio2, bio7, bio12, bio14, bio15, bio20** | **LH** | **1** | **0.8781±0.0149** | **0.0015** | **0.1096** |
| 2 | Same as above | LQP | 1 | 0.8623±0.0174 | 0.0046 | 0.1096 |
| 3 | Same as above | LQH | 2 | 0.8777±0.0150 | 0.0015 | 0.1142 |
| 4 | Same as above | LQH | 1 | 0.8801±0.0142 | 0.0031 | 0.1143 |
| 5 | Same as above | LQPH | 1.5 | 0.8808±0.0147 | 0.0015 | 0.1172 |
| 6 | Same as above | LQPH | 2 | 0.8782±0.0150 | 0.0015 | 0.1173 |
| 7 | Same as above | LQH | 1.5 | 0.8782±0.0148 | 0.0015 | 0.1187 |
| 8 | Same as above | LH | 1.5 | 0.8819±0.0145 | 0.0016 | 0.1205 |
| 9 | Same as above | LQPT | 1.5 | 0.8768±0.0151 | 0.0015 | 0.1219 |

Note: Variables’ full names (see table 1). L, Q, P, T and H are linear, quadratic, product, threshold and hinge features, respectively. RM is regularization multiplier, and SD is standard deviation. OR is test omission rate. Test AUC_cv_ is MaxEnt 10-fold cross-validation Area Under the ROC curve.

**Table S4.** Summary of performance statistics of *Phaseolus vulgaris* MaxEnt models. The best model is highlighted in bold.

| **ModelRank** | **Variables** | **MaxEnt settings** | | **Test AUC_cv_**  **(±SD)** | **OR** | |
| --- | --- | --- | --- | --- | --- | --- |
|  |  | **Features** | **RM** |  | **0%** | **10%** |
| **1** | **bio1, bio2, bio7, bio12, bio14, bio15, bio20** | **LQPH** | **2** | **0.8551±0.0238** | **0.0055** | **0.1023** |
| 2 | Same as above | LQH | 2 | 0.8520±0.0244 | 0.0083 | 0.1160 |
| 3 | Same as above | LQH | 1.5 | 0.8520±0.0237 | 0.0110 | 0.1161 |
| 4 | Same as above | LQPH | 1 | 0.8518±0.0239 | 0.0138 | 0.1189 |
| 5 | Same as above | LH | 1.5 | 0.8537±0.0239 | 0.0083 | 0.1242 |
| 6 | Same as above | LQPH | 1.5 | 0.8537±0.0239 | 0.0083 | 0.1242 |
| 7 | Same as above | LQPT | 2 | 0.8491±0.0238 | 0.0056 | 0.1324 |
| 8 | Same as above | LQH | 1 | 0.8532±0.0231 | 0.0164 | 0.1383 |
| 9 | Same as above | LQPT | 1.5 | 0.8511±0.024 | 0.0056 | 0.1573 |

Note: Variables’ full names (see table 1). L, Q, P, T and H are linear, quadratic, product, threshold and hinge features, respectively. RM is regularization multiplier, and SD is standard deviation. OR is test omission rate. Test AUC_cv_ is MaxEnt 10-fold cross-validation Area Under the ROC curve.


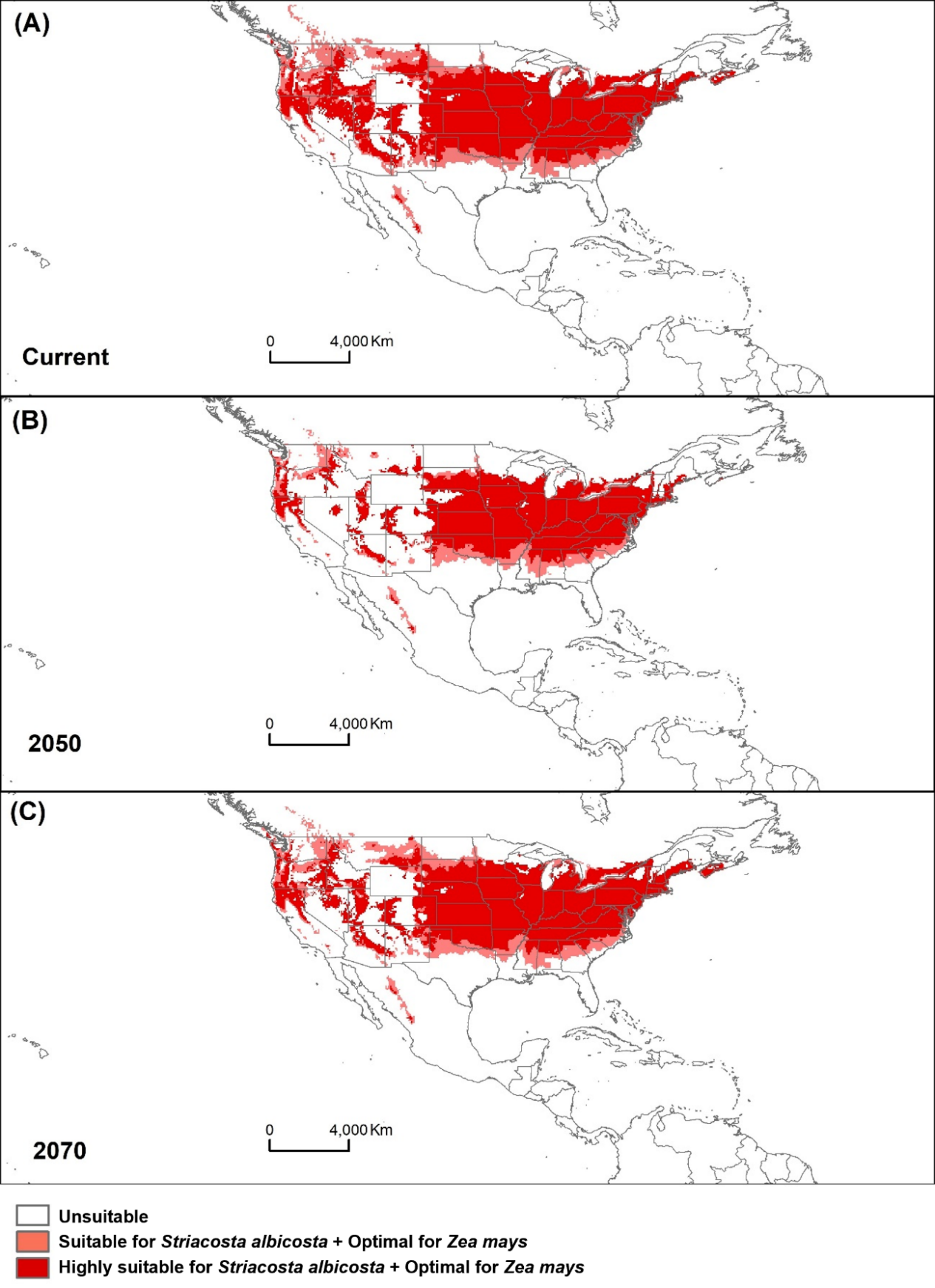


**Figure S1.** Habitat suitability in the USA under current and future climatic conditions in optimal areas for *Zea mays* cultivation with three suitability levels of *Striacosta albicosta*. Maps (A) current time, (B) 2050, and (C) 2070.


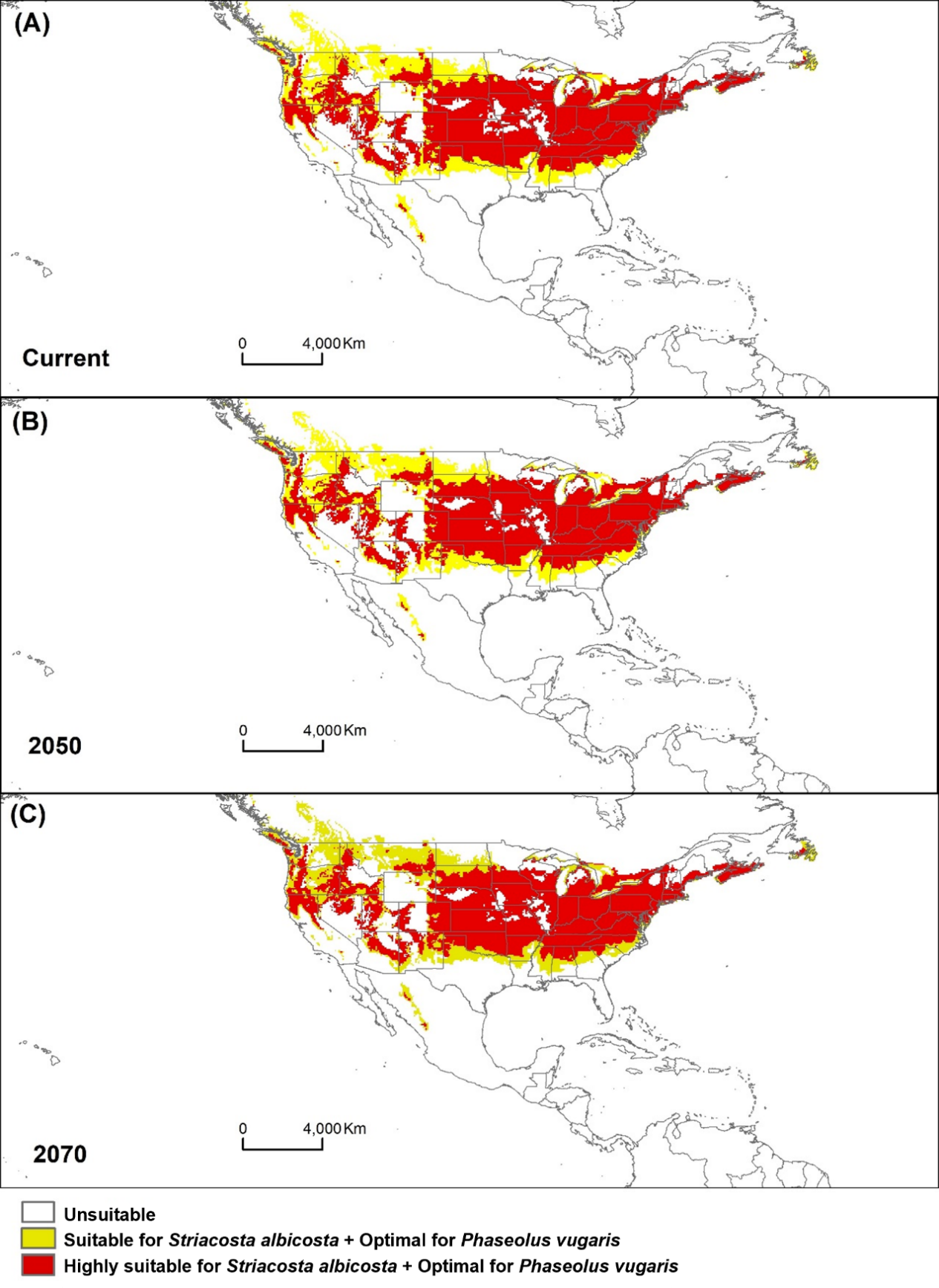


**Figure S2.** Habitat suitability in the USA under current and future climatic conditions in optimal areas for *Phaseolus vulgaris* cultivation with three suitability levels of *Striacosta albicosta*. Maps (A) current time, (B) 2050, and (C) 2070.


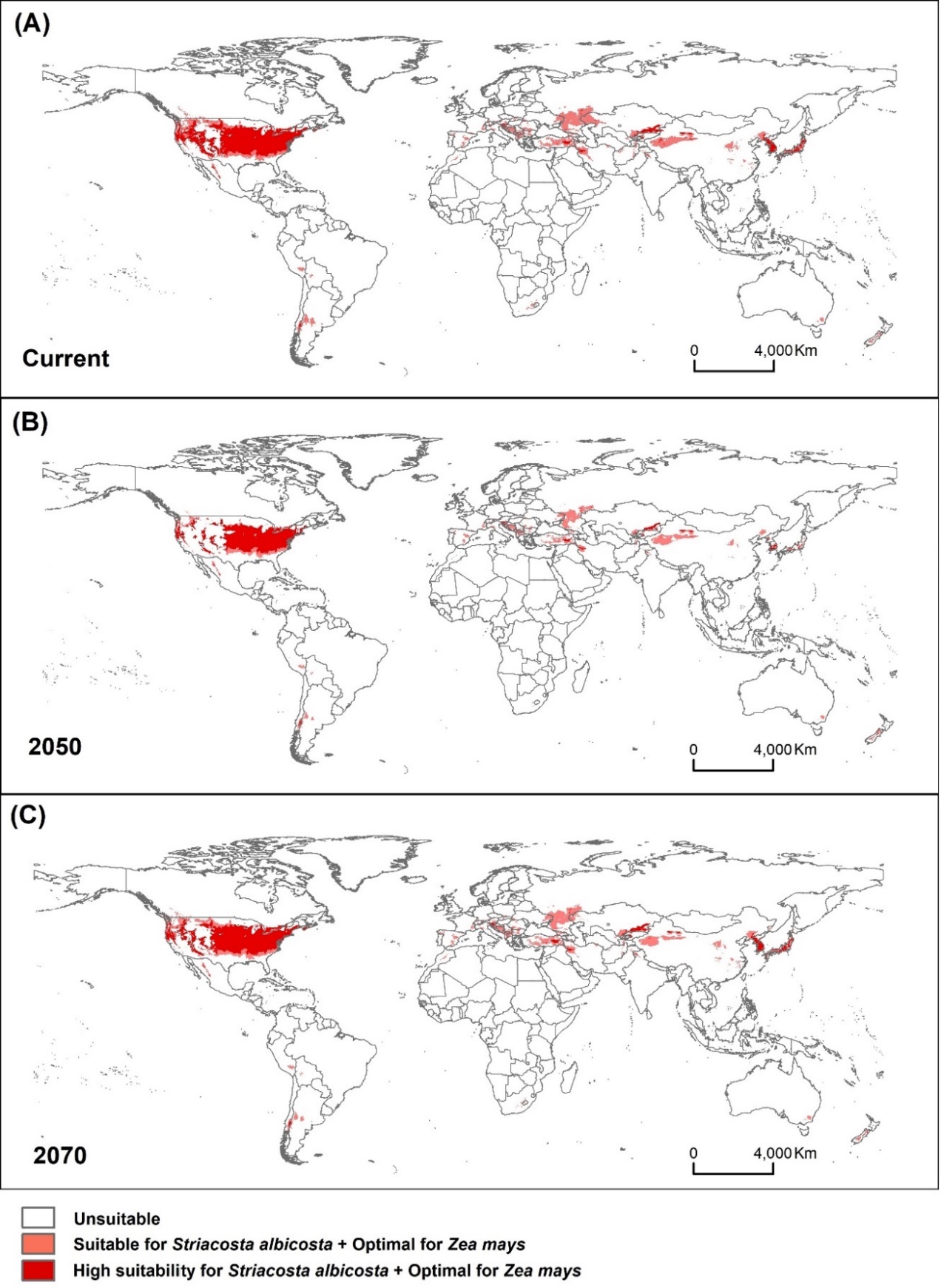


**Figure S3.** Habitat suitability under current and future climatic conditions in optimal areas for *Zea mays* cultivation with three suitability levels of *Striacosta albicosta*. Maps (A) current time, (B) 2050, and (C) 2070.

**
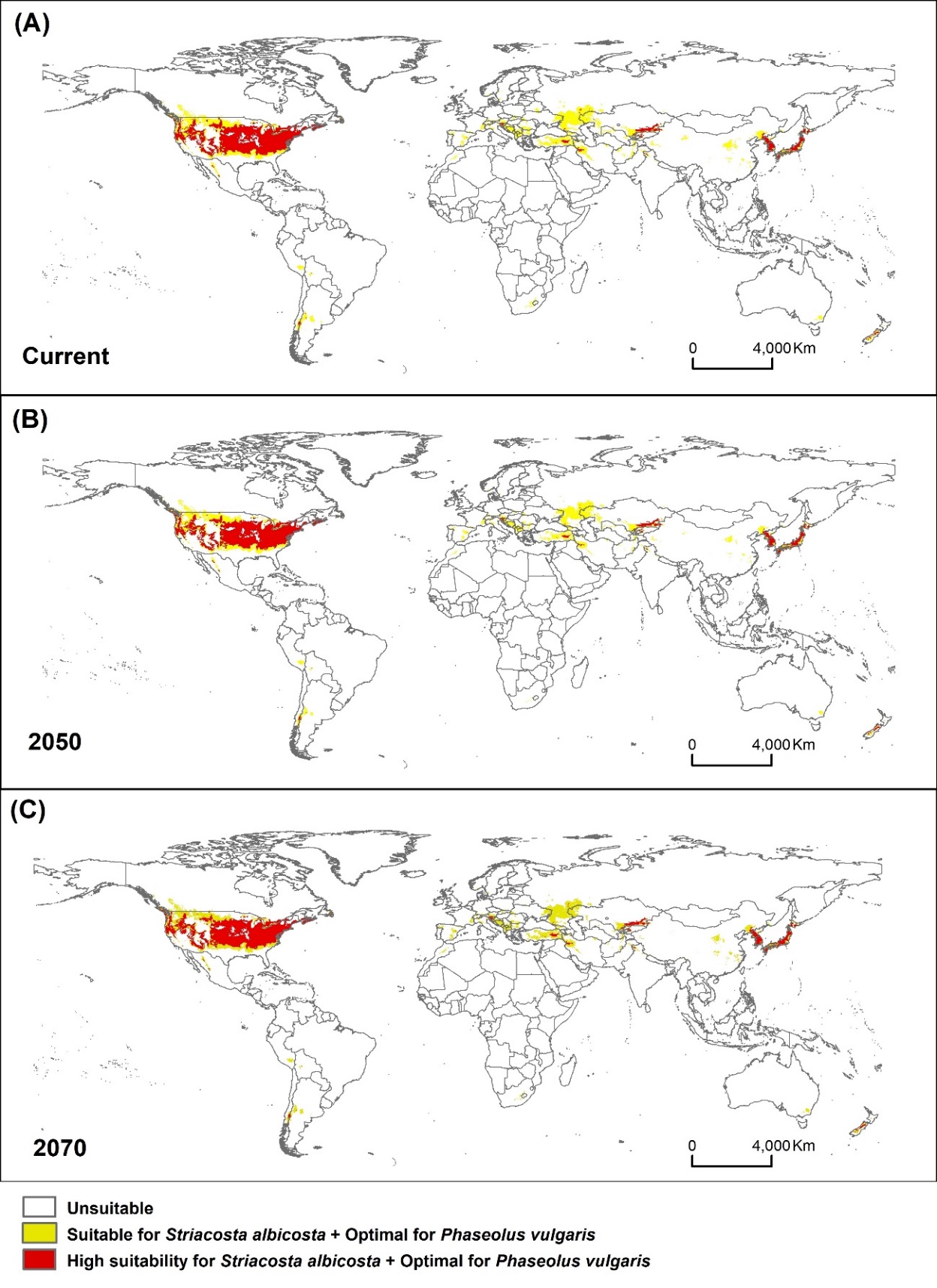
**

**Figure S4.** Habitat suitability under current and future climatic conditions in optimal areas for *Phaseolus vulgaris* cultivation with three suitability levels of *Striacosta albicosta*. Maps (A) current time, (B) 2050, and (C) 2070.


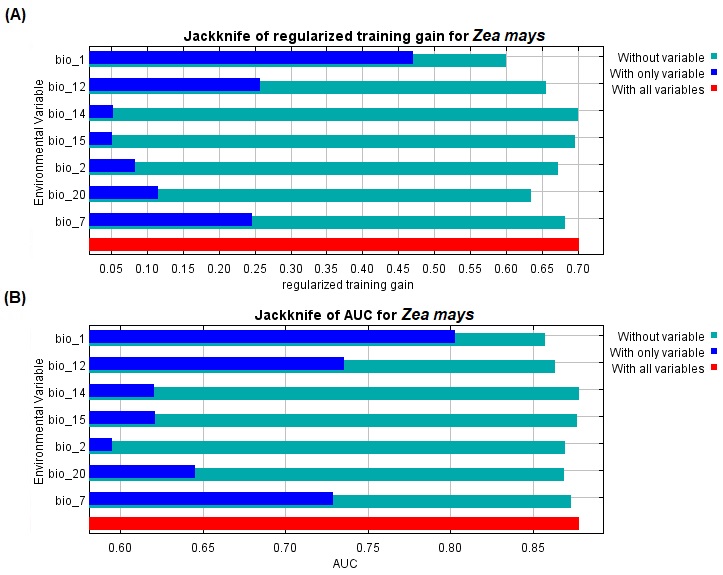


**Figure S5.** Relative importance of environmental variables based on the JackKnife test (A) Regularized training gain and (B) AUC in the *Zea mays* model.


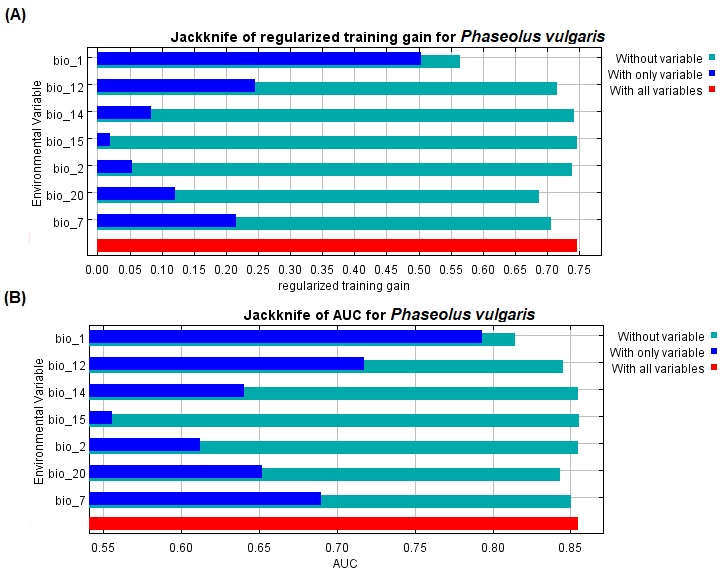


**Figure S6.** Relative importance of environmental variables based on the JackKnife test (A) Regularized training gain and (B) AUC in the *Phaseolus vulgaris* model.


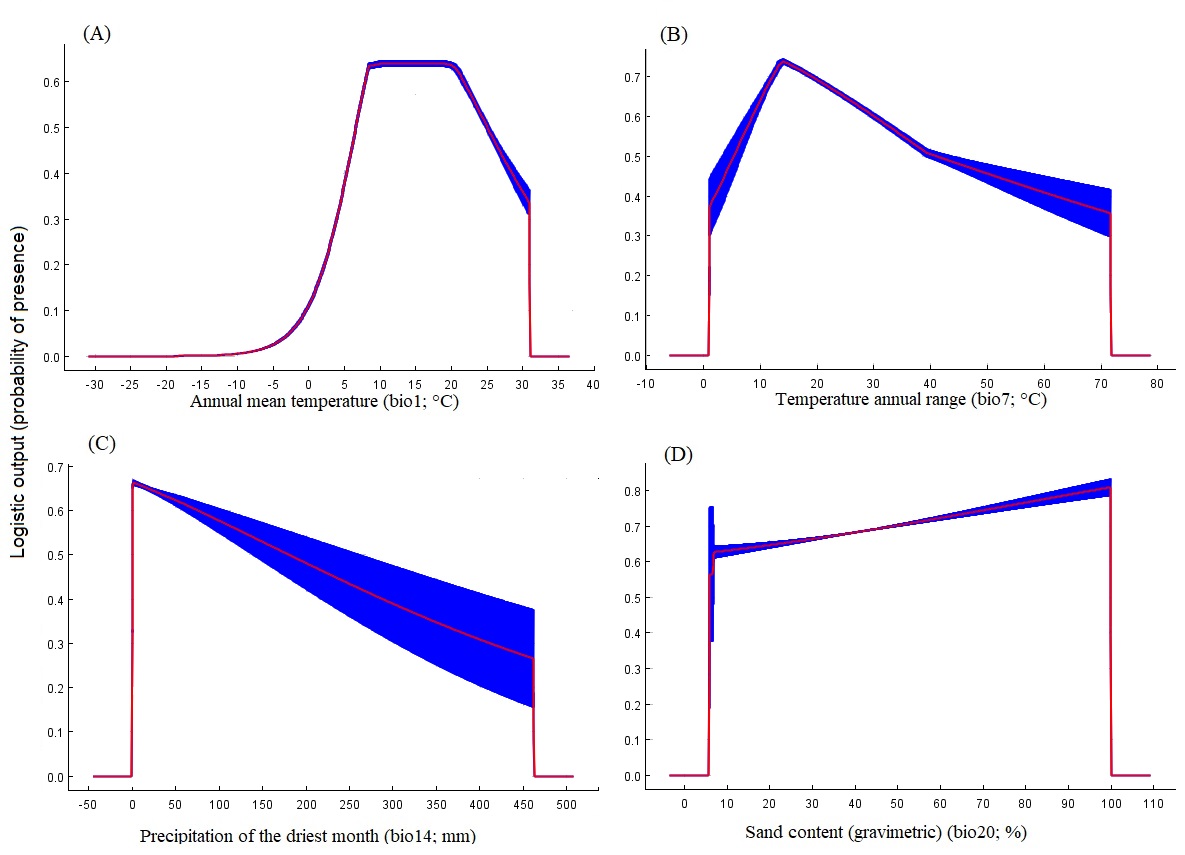


**Figure S7.** Response curves of the best predictors of *Zea mays* in the best model. (A) annual mean temperature (bio1; °C), (B) temperature annual range (bio7; °C), (C) Precipitation of the driest month (bio14; mm), and (D) Sand content (gravimetric) (bio20; %).


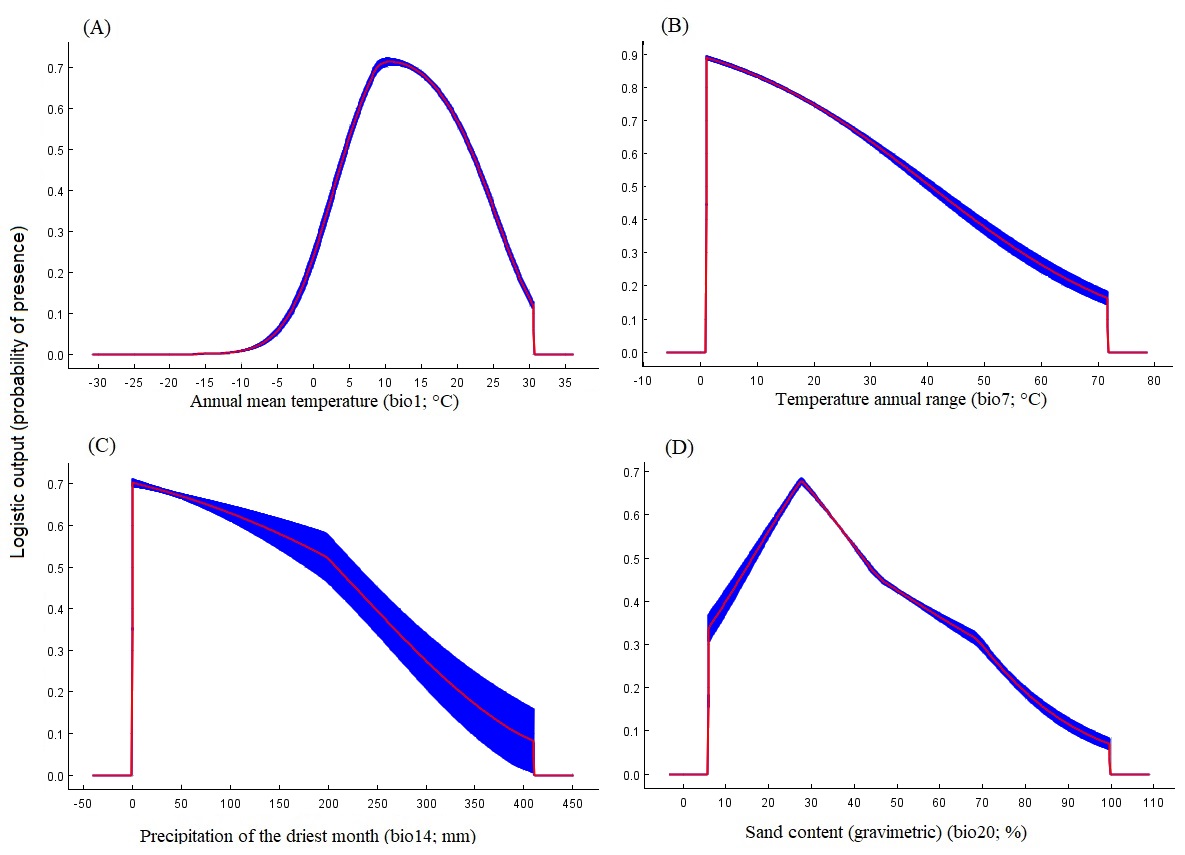


**Figure S8.** Response curves of the best predictors of *Phaseolus vulgaris* in the best model. (A) annual mean temperature (bio1; °C), (B) temperature annual range (bio7; °C), (C) Precipitation of the driest month (bio14; mm), and (D) Sand content (gravimetric) (bio20; %).
